# Vacuum dried membranes of poly (l-lactic acid) and bacterial cellulose for biomedical applications

**DOI:** 10.1186/1753-6561-8-S4-P50

**Published:** 2014-10-01

**Authors:** Drielle Justiniano de Souza, Luismar Marques Porto, Ana Paula Testa Pezzin

**Affiliations:** 1 Chemical Engineering Graduate Program, Federal University of Santa Catarina, Florianópolis, Santa Catarina, 88040623, Brazil; 2 Process Engineering Graduate Program, UNIVILLE, Joinville, Santa Catarina, 89219710, Brazil

## Background

Periodontitis is the inflammation of tissues supporting the tooth when gingivitis is not treated [1]. The treatment should reduce/eliminate inflammation of the tissue, which is induced by the biofilm with its byproducts and correct anatomic defects caused by the disease [2]. Guided tissue regeneration is a technique that applies membranes that form a blood clot that may allow the growth and differentiation of cells and regenerate the periodontal tissues. This should be able to block and isolate the area and contribute passively to tissue integration with guaranteed protection, including possible bacterial contamination [2,3]. This work aimed to obtain biocomposites of poly(l-lactic acid) (PLLA) with bacterial cellulose (BC) by different methodologies to be applied as membranes for the treatment of periodontitis. The characterization was performed by scanning electron microscopy, cell adhesion and contact angle. It was observed that the membranes 80/20 prepared by casting prior to vacuum drying of BC exhibited better adhesion. This membrane proved to be non-cytotoxic and more hydrophilic.

## Methods

Biocomposites of PLLA/BC were obtained from four different methods (M1-M4). M1: solvent exchange with BC; M2: Vacuum dried BC, with ratio of PLLA/BC 80/20; in M3 and M4 we used BC lyophilized membranes. In the third method PLLA/CHCl_3 _solution was poured over the BC membrane and in the fourth method PLLA/CHCl_3 _solution was filtered on a Gooch funnel. The evaluation of the composite membranes was performed by the techniques of scanning electron microscopy, cell adhesion (Vero cell line, ATCC CCL 81, passage 192) and contact angle.

## Results and conclusions

By visual inspection and the cell adhesion assay it was clear that the most satisfactory results were obtained for the membranes made with vacuum-dried BC/PLLA. On those membranes (M2 method), non-toxicity of the membrane was verified through the formation of a monolayer of adhered cells. Membranes obtained by this second method also showed more favorable results in decreasing the value of the contact angle, a consequence of the increasing hydrophilicity of the sample [5]. This membrane has a smooth surface and did not show toxicity to the cells by the addition of BC. These requirements are important for a biomaterial to be used for medical purposes. In this work BC and pure PLLA membranes were prepared and combined to form composites aimed to biomedical applications. Particular attention was given to the resulting biomaterial so as to guarantee that it could be used in the human body, therefore meeting the necessary initial requirements, such as non-cytotoxicity, biodegradability and bioresorption. Taking into account that PLLA is a biodegradable and bioresorbable polymer and BC is highly hydrophilic, through the analysis of cell adhesion and contact angle we showed that the second preparation method was the most appropriate, that is, the one using vacuum dried BC. The seeded cells formed a monolayer, indicating satisfactory adhesion; on the other hand, no cytotoxicity of the membrane and enhanced hydrophilicity of the composite membrane were observed, allowing the use of this membrane for biomedical applications.
